# The Effect of Minimally Invasive Hematoma Aspiration on the JNK Signal Transduction Pathway after Experimental Intracerebral Hemorrhage in Rats

**DOI:** 10.3390/ijms17050710

**Published:** 2016-05-13

**Authors:** Haitao Pei, Tao Jiang, Guofang Liu, Zhaoxing Li, Kai Luo, Jingjiao An, Guangcheng Li, Yunliang Guo

**Affiliations:** 1 Department of Neurology, Affiliated Hospital of Qingdao University, Qingdao 266003, Shandong, China; peihtao@163.com (H.P.); guofangliu1123@sina.com (G.L.); 2 Department of Emergency, Linyi People’s Hospital, Linyi 276003, Shandong, China; fakewings2012@163.com; 3 Institute of Integrative Medicine, Qingdao University, Qingdao 266003, Shandong, China; 15854207702@163.com (Z.L.); 15064888813@163.com (K.L.); 4 Department of Radiology, Affiliated Hospital of Qingdao University, Qingdao 266003, Shandong, China; anjgjo@163.com; 5 Beijing Meidehoupu Science and Technology Company Limited, Beijing 102206, China; 15005326618@163.com; 6 Institute of Cerebrovascular Diseases, Affiliated Hospital of Qingdao University, Qingdao 266003, Shandong, China

**Keywords:** minimally invasive hematoma aspiration, intracerebral hemorrhage, brain edema, JNK, rats

## Abstract

Objective: To explore the effect of minimally invasive hematoma aspiration (MIHA) on the c-Jun NH_2_-terminal kinase (JNK) signal transduction pathway after intracerebral hemorrhage (ICH). Methods: In this experiment, 300 adult male Wistar rats were randomly and averagely divided into sham-operated group, ICH group and MIHA group. In each group, 60 rats were used in the detection of indexes in this experiment, while the other 40 rats were used to replace rats which reached the exclusion criteria (accidental death or operation failure). In ICH group and MIHA group, ICH was induced by injection of 70 µL of autologous arterial blood into rat brain, while only the rats in MIHA group were treated by MIHA 6 h after ICH. Rats in sham-operated group were injected nothing into brains, and they were not treated either, like rats in ICH group. In each group, six rats were randomly selected to observe their Bederson’s scales persistently (6, 24, 48, 72, 96, 120 h after ICH). According to the time they were sacrificed, the remaining rats in each group were divided into 3 subgroups (24, 72, 120 h). The change of brain water content (BWC) was measured by the wet weight to dry weight ratio method. The morphology of neurons in cortex was observed by the hematoxylin–eosin (HE) staining. The expressions of phospho-c-Jun NH_2_-terminal kinase (pJNK) and JNK in peri-hematomal brain tissue were determined by the immunohistochemistry (IHC) and Western blotting (WB). Results: At all time points, compared with the ICH groups, the expression of pJNK decreased obviously in MIHA groups (*p* < 0.05), while their Bederson’s scales and BWC declined, and neuron injury in the cortex was relieved. The expression level of JNK was not altered at different groups. The data obtained by IHC and WB indicated a high-level of consistency, which provided a certain dependability of the test results. Conclusion: The JNK signal transduction pathway could be activated after intracerebral hemorrhage, with the expressions of pJNK increasing. MIHA could relieve the histo-pathological damage of nerve cells, reducing brain edema and neurological deficits, and these neuroprotective effects might be associated with suppression of JNK signal transduction pathway.

## 1. Introduction

Intracereral hemorrhage (ICH), with poor prognosis, high mortality, and disability rate, is a common and frequently-occurring disease, accounting for 9%–27% in all types of stroke. Though various forms of active treatment have been given in clinic, the mortality rate of ICH still remains stubbornly high owing to the absence of specific therapy [1,2,3]. In addition to medical treatment and traditional surgical therapy (craniotomy), minimally-invasive hematoma aspiration (MIHA, also known as the stereotactic aspiration technique) is being given attention recently. Compared with traditional surgery treatment, MIHA, as a therapy aiming at removing intracranial hematoma, cannot only eliminate the space occupying effect certainly, but also have advantages of less surgical trauma and better recuperation, although there is no significant impact on mortality [3,4,5].

The c-Jun NH_2_-terminal kinases (JNKs) are components of the mitogen-activated protein kinases (MAPKs), which were found as a protein-serine/threonine kinase at 1993. JNKs are nearly all either 46 or 54 kDa and mainly distributed in cytoplasm, which is also known as stress-activated kinase (SAPK) [6]. JNKs are quickly activated then change the expressions of relevant genes to control cell proliferation, differentiation, and apoptosis, under the stimulus of external factors, such as growth factor, cytokine, inflammatory factor, *etc.* [7]. The JNK signal transduction pathway takes part in occurrence and development of central nervous system disease, especially in Alzheimer disease (AD), Parkinson disease (PD), and cerebral ischemia [8]. Moreover, other studies have suggested that the JNK signal transduction pathway plays a role in ICH [9].

Research has suggested that early removal of the hematoma by MIHA could reduce the expansion of the hematoma, the production of inflammatory mediator, and the direct chemical damage to brain tissue [10]. However, the research on the relationship between MIHA and JNK pathway has not been reported. This experiment is determined to explore and investigate the influence of MIHA on the JNK signal transduction pathway and its neuroprotective effect, while the method of injecting autologous arterial blood into the rat brain is used to induce the model of ICH.

## 2. Results

### 2.1. Neurological Evaluation

**Figure 1:**
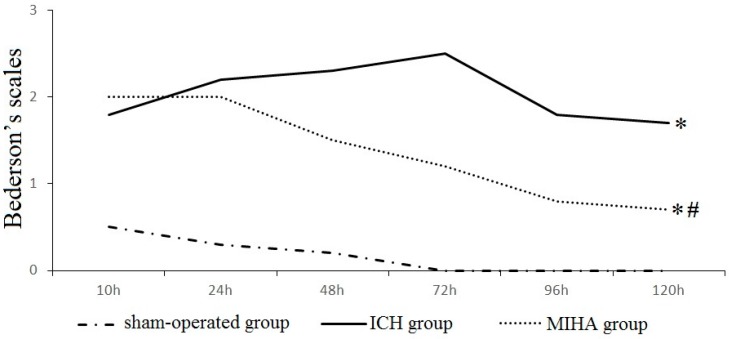
The Bederson’s scales of different groups at each time point. * *p* < 0.05 *vs.* the sham-operated group, # *p* < 0.05 *vs.* the intracereral hemorrhage (ICH) group.

There were no obvious neuromotor dysfunction on rats in the sham-operated group, while different degrees of neurological deficits were seen in ICH group and MIHA group at each time point. When tested at 6 and 24 h after ICH, Bederson’s scales in ICH group and MIHA group were obviously higher than those in the sham-operated group (*p* < 0.05), while there was no significant difference between ICH group and MIHA group (*p* > 0.05). When tested at 48, 72, 96, and 120 h, the Bederson’s scales in ICH group were the highest, followed by MIHA group’s, while those were the lowest in sham-operated group, difference among them was significant (*p* < 0.05) (Table 1 and Figure 1). The results show that after treated by MIHA the Bederson’s scales of rats decrease markedly at the time points 48, 72, 96, and 120 h after ICH, compared with ICH group, which means MIHA can significantly improve the neurological deficits. A subtle and opposite result is determined 6 h after ICH, but it turns out to be statistically insignificant. This may be related to the premature detection, as any kind of treatment should take a certain period of time to come into effect.

### 2.2. Brain Water Content (BWC)

When tested at 24, 72 and 120 h after ICH, the brain water contents (BWCs) of ICH group were the highest, followed by MIHA group’s, while those of sham-operated group were the lowest, difference among them was significant (*p* < 0.05) (Table 2). The results show that MIHA can significantly reduce the BWC of rats, thus, relieving brain edema after ICH.

### 2.3. Effects on Histopathology

**Figure 2:**
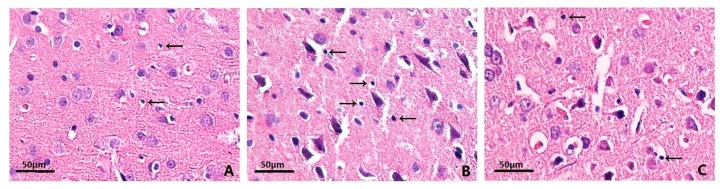
The morphological and structural changes of neurons in different groups, by HE staining, magnification 400×, scale bar = 50 µm. (**A**) Sham-operated group; (**B**) ICH group; (**C**) MIHA group; (arrows) denatured cells.

Hematoxylin–eosin (HE) staining revealed that rats in sham-operated group had clear layers of brain tissue structures, stained evenly and with a regular cell shape, compared with rats in ICH group and MIHA group. In ICH group, irregular arrangement, disorganization, and karyopyknosis of the nerve cells were identified with hyperchromatic nuclei. The neuronal necrosis and degeneration in MIHA group were less serious than that in ICH group (Figure 2). The denatured cell index (DCI) was adopted to show the injury severity of neurons, which was calculated as “denatured cell counts/total cell counts”. When tested at 72 h after ICH, the DCIs of ICH group were the highest, followed by MIHA group’s, while those of sham-operated group were the lowest, the difference among them was significant (*p* < 0.05) (Table 3). This evidence shows that the histopathological damage of nerve cells is obviously reduced after being treated by MIHA.

### 2.4. Changes on Immunohistochemistry

**Figure 3:**
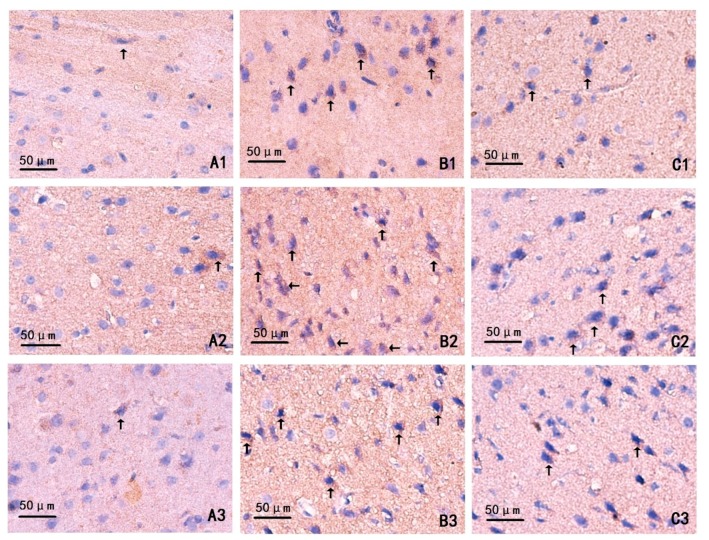
The expression of phospho-c-Jun NH_2_-terminal kinase (pJNK) in cortex of rats shown by immunohistochemical assay, magnification 400×, scale bar = 50 µm. (**A1**–**A3**) Sham-operated groups (24 h subgroup, 72 h subgroup, and 120 h subgroup); (**B1**–**B3**) ICH groups (24 h subgroup, 72 h subgroup, 120 h subgroup); and (**C1**–**C3**) MIHA groups (24 h subgroup, 72 h subgroup, 120 h subgroup); (arrows) positive cells.

**Figure 4:**
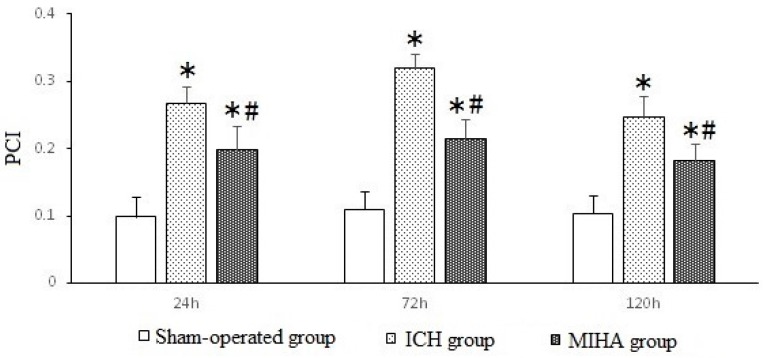
The positive cell indexs (PCIs) of pJNK in different groups at each time point shown by immunohistochemical assay. * *p* < 0.05 *vs.* the sham-operated group, # *p* < 0.05 *vs.* the ICH group.

At different time points, the cells in the sham-operated group were gently dyed, the number of positive cells was small, and the expression of phospho-c-Jun NH_2_-terminal kinase (pJNK) was weaker, compared with the ICH group and MIHA group (Figure 3). The positive cell index (PCI) was adopted to show the expression levels of pJNK, which was calculated as “positive cell counts/total cell counts”. When tested at 24, 72, and 120 h after ICH, the PCIs of ICH group were the highest, followed by MIHA group’s, while those of the sham-operated group were the lowest, difference among them was significant (*p* < 0.05) (Table 4 and Figure 4). It shows that the JNK signal transduction pathway can be activated after intracerebral hemorrhage, and that MIHA can significantly reduce the expression of pJNK, thus, depressing this signal transduction pathway.

### 2.5. Western Blotting

**Figure 5:**
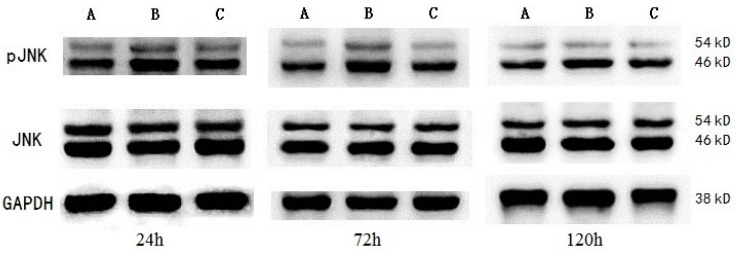
The expressions of pJNK and c-Jun NH_2_-terminal kinase (JNK) in peripheral region of hematoma at each time point, shown by Western blotting. (**A**) Sham-operated group; (**B**) ICH group; and (**C**) MIHA group.

**Figure 6:**
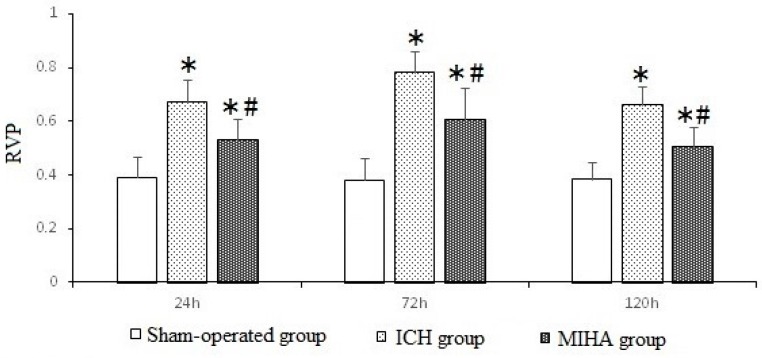
The relative value of the proteins (RVPs) of pJNK at each time point determined by Western blotting. * *p* < 0.05 *vs.* the sham-operated group, # *p* < 0.05 *vs.* the ICH group.

The results of protein expression were quantified by the relative value of the protein (RVP), which was calculated as “the gray value of target protein bands/the gray value of internal control bands”. When tested at 24, 72, and 120 h after ICH, the pJNK expression of ICH group was the highest, followed by MIHA group’s, while that of sham-operated group was the lowest, difference among them was significant (*p* < 0.05) (Table 5 and Figure 5 and Figure 6), which means the expression of pJNK was obviously decreased after MIHA. However, the expression of total JNK did not change, as there were no significant differences among the three groups for the RVP of total JNK at the three time points (*p* > 0.05). These results suggest that MIHA can significantly inhibit the JNK signal transduction pathway by reducing the expression of pJNK rather than that of total JNK.

## 3. Discussion

ICH is a common and frequently-occurring disease with poor prognosis, high mortality, and disability rate. In this experiment, we used the method of injecting autologous arterial blood into the rat brain to induce the model of ICH. The injection volume of blood (70 µL) is a frequent intracereral hemorrhage volume in clinical practice after the conversion (1 µL in rats is equivalent to 0.75 mL in human [11]), and the aspiration volumes of rats reaching the inclusion criteria in practice are 20~50 µL, which means hematoma and is effectively eliminated.

The JNK signal transduction pathway takes part in the process of inflammation, stress, cell growth, and death. When the extracellular environment changes, MAPK kinase 4 (MKK4), and MAPK kinase 7 (MKK7) are activated and they phosphorylate JNK on Tyr182 and Thr180 ulteriorly to cooperate to activate JNK *in vivo* [12]. Lots of evidence have shown that the JNK signal transduction pathway involve in occurrence and development of neurological diseases [8,9]. In the research of the middle cerebral artery occlusion models of adult rats and mice, scientists found that ischemia-reperfusion injury can mediate the activation of JNK3 and the JNK peptide inhibitor D-JNKI-1 can exert different degrees of neuroprotective effects [13]. Wang *et al.*’s [14] study showed that the JNK signal transduction pathway was activated after ICH, while intravenous injection of the JNK peptide inhibitor D-JNKI-1 could alleviate cerebral edema and improve the neurological deficits via reducing the transcriptional activity of c-Jun.

In our study we demonstrated that JNK was activated following ICH, which might induce inflammation and apoptosis, thus leading to brain edema and neurological deficits. After treated by MIHA, the histopathological damage of nerve cells relieved, Bederson’s scales and BWC declined. These beneficial effects might be associated with inhibition of JNK signal transduction pathway (the expression of pJNK decreased).

Now it has been confirmed that an inflammatory response occurs in and around the hematoma after ICH, which is characterized by the activation of microglia and inflammatory cell (especially neutrophils and macrophages) infiltration, and that infiltrating leukocytes and activated microglia may release cytotoxic mediators contributing to secondary brain injury [15]. JNKs are expressed in microglias, oligodendrocytes, astrocytes, the microglial activation and the release of inflammatory cytokines can phosphorylate JNK leading to activation of transcription factors, such as c-Jun and NFκB, while the JNK inhibitor SP600125 can reduce the of synthesis inflammatory cytokines in microglias [16,17,18]. Thus, it can be seen that JNKs have a connection with inflammation of brain tissue after ICH. Meanwhile, the components of hematoma, including thrombin, hemoglobin, and iron, can cause inflammatory cell infiltration, cell death, and brain edema in the peri-hematomal area [19,20,21,22,23]. Multiple lines of evidence indicate that thrombin is a central event in the mechanisms of ICH [24]. And argatroban as a direct thrombin inhibitor can decline the phosphorylation level of JNK in peri-hematomal area in part after ICH, which demonstrate that the release of thrombin plays a role in the activation of JNK signal transduction pathway [25]. Huang *et al.* [26] reported that lysed erythrocytes and hemoglobin were essential parts in brain edema after ICH. On the other hand, research showed that an intracerebral infusion of ferrous iron increased the expression level of activated-JNK in brain and systemic administration of deferoxamine suppressed this upregulation [27].

Combining the above facts and our experimental results, we speculate that by removing the intracranial hematomas, MIHA can not only eliminate the space occupying effect, but also decrease the contents of local stimulating factors (thrombin, hemoglobin, iron, *etc.*), thereby possibly suppressing the JNK signal transduction pathway, and then reducing the inflammatory reaction and playing a role in neuroprotection.

In addition, the neuronal apoptosis is also important in the pathophysiologic process of ICH. The JNK expression is closely related with neuronal apoptosis, which can be suppressed by inhibiting the activation of JNK [28,29]. Lai *et al.* [30] reported that using the JNK inhibitor SP600125, or using SP600125 and z-VAD together could significantly suppress the neuronal apoptosis in heroin-induced spongiform leukoencephalopathy. He *et al.*’s study [31] showed that (−)-Epigallocatechin-3-gallate (EGCG) could significantly prevent apoptosis by suppressing JNK phosphorylation, and that the JNK inhibitor SP600125 could reduce thrombin-induced caspase 3 activation and apoptosis after ICH. Zhang *et* *al.* [32] reported that ICH led to upregulation of apoptosis-related genes in the brain tissues, and that MIHA could down-regulate the expression of Hsp70 and Bax induced by ICH, but upregulates the expression of Bcl-2. Therefore, we speculate that MIHA may suppress neuronal apoptosis by inhibiting the JNK signal transduction pathway.

## 4. Materials and Methods

### 4.1. Animals

A total of 300 adult healthy male specific pathogen-free (SPF)-grade Wistar rats, weighing 230–260 g, were provided by the Qingdao Experimental Animal Center (SCXK [LU] 20140001). All the rats were housed in the laboratory at room temperature (25 ± 2 °C) and they had free access to food and water under natural illumination during an acclimatization period of seven days before the experiment beginning. The study protocol was approved by the Ethics Committee of Qingdao University Medical College (QUMC 2011-09).

### 4.2. Experiment Groups

These 300 adult male Wistar rats were randomly and averagely divided into the sham-operated group, ICH group, and MIHA group. In each group, 60 rats were used in the detection of indexes in this experiment, while the other 40 rats were used to replace rats which reached the exclusion criteria (accidental death or operation failure). In ICH group and MIHA group, ICH was induced by injection of 70-µL autologous arterial blood into rat brain, while only the rats in the MIHA group were treated by MIHA 6 h after ICH. Rats in the sham-operated group were injected nothing into their brains, and they were not treated either, like rats in the ICH group. In each group, six rats were randomly selected to observe their Bederson’s scales persistently (6, 24, 48, 72, 96, and 120 h after ICH). According to the time they were sacrificed, the remaining rats in different groups were divided into three subgroups (24, 72, 120 h, each group has 18 rats). In each subgroup, six rats were randomly selected to evaluate the brain water content (BWC) by the wet weight to dry weight ratio method, six rats were randomly selected for preparation of paraffin section which would be used in the hematoxylin–eosin (HE) staining and immunohistochemistry (IHC), and the other six rats would be used for preparation of protein samples in Western blotting (WB). All of the groups and subgroups were divided by random number table.

### 4.3. Induction of ICH and Treatment Methods

The ICH model in rats was built by the method which had been described in the study of Yang *et al.* [33]. First of all, rats were anesthetized by intraperitoneal injection of 10% chloral hydrate according to the dose of 400 mg/kg, and placed on the three-dimensional stereotactic frame (Jiangwan type I–C, Shanghai Precision Instrument Co., Ltd., Shanghai, China) in prone position. The scalp was incised longitudinally in midline, and a 2-mm burr hole was made in the skull by a dental drill (204-SH37LN, Saeshin Precision Ind., Co., Taegu, Korea). Fresh autologous blood was got from the severed tail artery and drawn into a 100-µL microsyringe which was then lowered vertically through the burr hole into the caudate nucleus of right brain (3.0 mm lateral right to the sagital suture, 0.2 mm anterior to the bregma, and 5.8 mm deep below burr hole) immediately. Then 70-µL blood was injected into the rat brain slowly at a rate of about 10 µL/min and the microsyringe was indwelt for 20 min. In the end, the microsyringe was removed slowly, and the incisions in the skin were closed. The Bederson’s scale [34] was used to evaluate whether the models were successful or failed (≥1 means successful) 6 h later. The successfully established rats models in MIHA groups were anesthetized and placed on the three-dimensional stereotactic frame again. Then 5-µL urokinase solutions (prepared with urokinase for injection and physiological saline, 3000 U: 5 µL, Nanjing Nanda Pharmaceutical. Co., Ltd., Nanjing, China) was injected into the right brain by a 10-µL microsyringe at the same coordinates (3.0 mm lateral right to the sagital suture, 0.2 mm anterior to the bregma, and 5.8 mm deep below the burr hole). The 10-µL microsyringe was replaced by another 100-µL microsyringe to aspirate hematoma 2 h later. The aspiration volumes were recorded. Rats in sham-operated groups went a sham procedure (lower microsyringe vertically into the right brain at the same coordinates, without injecting arterial blood into caudate nucleus, Bederson’s scale = 0). Exclusion criteria included accidental death, the failure in animal models making and aspiration volumes <20 µL. They were subsequently excluded from the study and replaced by the same batch of rats which reached the qualified standard. All the rats were put back to the experimental animal house with free access to food and water.

### 4.4. Neurological Evaluation

The Bederson’s scales were evaluated persistently by an observer who was blinded to the study at five time points (24, 48, 72, 96, 120 h after ICH).

### 4.5. Brain Water Content (BWC)

To evaluate BWC, the wet weight to dry weight ratio method was used. After being sacrificed at corresponding time points, rats in different subgroups were decapitated to get the brains rapidly. Filter paper was used to blot up liquid from the surface of brains and a piece of tissue sample in the peripheral region of hematoma was cut off from basal ganglia immediately. On an electronic analytical balance, this piece of tissue sample was weighed first time to record the wet weigh. Then the tissue sample was dried in an oven at 95 °C for 24 h and reweighed to record the dry weight. The BWC = (wet weight − dry weight)/wet weight × 100%.

### 4.6. Preparation of Paraffin Section

Six rats were selected from each subgroup and anesthetized by intraperitoneal injection of 10% chloral hydrate according to the dose of 400 mg/kg. Before they were sacrificed at corresponding time points (24, 72, and 120 h), physiological saline and 4% formaldehyde solution were administered to perform cardiac perfusion fixation. After that the rats were decapitated to get brains. Ethanol, xylene, and paraffin were separately used for dehydration, vitrification, and embedding in sequence. Serial coronal sections were put on microscopic slides and stored until further detection, after treated with poly-l-Lysine.

### 4.7. Hematoxylin–Eosin (HE) Staining

After routine deparaffinage, the sections of those six rats mentioned above were washed with double-distilled water and stained with hematoxylin for 5 min. The color was separated with 1% hydrochloric acid alcohol for 20 s. Then the section were dipped in 1% ammonia water for 30 s, dyed with eosin for 5 min. Alcohol, dimethylbenzene, and neutral gum were separately used for dehydration, hyalinization, and seal in sequence. Six non-overlapping views of the cortex in each section were randomly chosen to enumerate the denatured cell counts and total cell counts, under a microscope at 400× magnification. The average was calculated to show the result of each rat. The DCI was adopted to show the injury severity of neurons, which was calculated as “denatured cell counts/total cell counts”. All the DCIs of these six rats were calculated and presented as mean ± standard deviation ($\bar{x}$ ± s).

### 4.8. Immunohistochemistry Staining (IHC)

The paraffin sections of those six rats were de-waxed and washed as above. According to the manufacturer’s instructions, the immunohistochemical procedures were performed strictly. Rabbit anti-rat pJNK monoclonal antibody (1:50, 4668S, Cell Signaling Tech. Co., Ltd., Danvers, MA, USA) was used as the primary antibody. The sections were colored by 3,3′-diaminobenzidine (DAB) and observed at 400× magnification. Those sections in which 0.01 mol/L phosphate buffered saline (PBS) replaced the primary antibody were regard as negative control and they exhibited no positive responses, while the positive cells appeared with brown granules. Six non-overlapping views of the cortex from four serial slices were randomly selected to enumerate the positive cell counts and total cell counts. The average was calculated to show the result of each rat. The PCI was adopted to show the expression levels of pJNK, which was calculated as “positive cell counts/total cell counts”. All the PCIs of these six rats were calculated and presented as $\bar{x}$ ± s.

### 4.9. Preparation of Protein Samples

The six residual rats in each subgroup were anesthetized by intraperitoneal injection of 10% chloral hydrate (400 mg/kg, intraperitoneally) according to the dose of 400 mg/kg. Before they were decapitated at corresponding time points (24, 72, and 120 h), 300-mL physiological saline were administered to perform cardiac perfusion. 100-mg brain tissue from each rat was put into 1.5-mL Eppendorf tubes. RIPA lysis buffer (P0013B, Beyotime Biotechnology Co., Ltd., Shanghai, China) was added at a proportion of 10 mg:100 µL. The cortical tissues in Eppendorf tubes were ground in a 4 °C ice bath and shaken gently for 30 min to lyse completely. After 10,000 r/min centrifugation for 20 min at 4 °C, the supernatant was collected into a new Eppendorf tubes and its protein content was determined by a BCA protein assay kit (P0010, Beyotime Biotechnology Co., Ltd., Shanghai, China). The protein samples were mixed with 5× sodium dodecyl sulfate polyacrylamide gel electropheresis (SDS-PAGE) sample loading buffer at a proportion of 4:1, afterwards boiled at 95~97 °C, and stored at −80 °C.

### 4.10. Western Blotting (WB)

After separation by 12% SDS-PAGE, the protein samples were transferred onto polyvinylidene fluoride membranes, which would be subsequently blocked with 5% nonfat dry milk in Tris-buffered saline at room temperature for 2 h. As required, the blocked membranes were, respectively, incubated with the primary antibodies of pJNK (1:1000, 4668S, Cell Signaling Tech. Co., Ltd., Danvers, MA, USA), JNK (1:1000, 9252S, Cell Signaling Tech. Co., Ltd., Danvers, MA, USA), or GAPDH (1:1000, bsm-0978M, Beijing Biosynthesis Biotechnology Co., Ltd., Beijing, China) overnight at 4 °C. The next day, the membranes were incubated with goat anti-rabbit HRP-conjugated secondary antibody (1:5000, BA1054, Boster Bioengineering Co., Ltd., Wuhan, China) or goat anti-mouse HRP-conjugated secondary antibody (1:5000, BA1050, Boster Biotechnology) for 1 h at room temperature. Protein bands were visualized using Immobilon™ Western chemiluminescent HRP substrate (WBKLS0500, Millipore, MA, USA), while images were obtained by a BioSpectrum 810 imaging system (Ultra-Violet Products Ltd., Upland, CA, USA). The signals were quantified by RVP, which was calculated as “the gray value of target protein bands/the gray value of internal control bands”. In this study, the gray value of protein bands was measured by Image-Pro Plus 6.0 (Media Cybernetics Inc., Bethesda, MD, USA) software, and GAPDH served as internal control. The results were presented as $\bar{x}$ ± s.

### 4.11. Statistical Method

All the results were expressed as $\bar{x}$ ± s. For Bederson’s scales (ranked data), multi-group comparisons were made with Kruskal-Wallis test, and two-group comparisons were made with Nemenyi test. For the other data, multi-group comparisons were performed with one-way analyses of variance (one-way ANOVA), and two-group comparisons were performed with LSD-*t* test. A difference was considered significant when *p* < 0.05.

## 5. Conclusions

In summary, our findings suggest that the JNK signal transduction pathway could be activated after intracerebral hemorrhage with the expression of pJNK increasing, and that MIHA could decrease the expression of pJNK, thus relieving the histopathological damage on nerve cells, reducing brain edema and neurological deficits. These neuroprotective effects might be associated with suppression of inflammation and apoptosis induced by the inhibition of the JNK signal transduction pathway.

## Figures and Tables

**Table 1:** The Bederson’s scales in different groups at each time point ($\bar{x}$ ± s).

Groups	*n*	6 h	24 h	48 h	72 h	96 h	120 h
Sham-operated group	6	0.5 ± 0.5	0.3 ± 0.5	0.2 ± 0.4	0.0 ± 0.0	0.0 ± 0.0	0.0 ± 0.0
Intracereral hemorrhage (ICH) group	6	1.8 ± 0.8 *	2.2 ± 0.8 *	2.3 ± 0.5 *	2.5 ± 0.5 *	1.8 ± 0.8 *	1.7 ± 0.5 *
Minimally invasive hematoma aspiration (MIHA) group	6	2.0 ± 0.9 *	2.0 ± 0.6 *	1.5 ± 0.5 *^,#^	1.2 ± 0.8 *^,#^	0.8 ± 0.4 *^,#^	0.7 ± 0.5 *^,#^
χ^2^		8.70	11.13	13.56	13.95	13.65	13.01
*P*		0.01	0.00	0.00	0.00	0.00	0.00

* *p* < 0.05 *vs.* the sham-operated group, # *p* < 0.05 *vs.* the ICH group.

**Table 2:** The brain water contents (BWCs) of rats in different groups at each time point ($\bar{x}$ ± s, %).

Groups	*n*	24 h	72 h	120 h
Sham-operated group	6	72.205 ± 0.676	72.613 ± 0.990	72.328 ± 0.802
ICH group	6	80.418 ± 0.787 *	84.369 ± 1.009 *	82.198 ± 0.896 *
MIHA group	6	77.624 ± 1.003 *^,#^	80.266 ± 0.928 *^,#^	79.664 ± 1.113 *^,#^
*F*		150.70	223.90	176.20
*P*		0.00	0.00	0.00

* *p* < 0.05 *vs.* the sham-operated group, # *p* < 0.05 *vs.* the ICH group.

**Table 3:** The denatured cell indexs (DCIs) s in different groups shown by hematoxylin–eosin (HE) staining 72 h after intracereral hemorrhage (ICH) ($\bar{x}$ ± s).

Groups	*n*	72 h denatured cell index (DCI)
Sham-operated group	6	0.160 ± 0.052
ICH group	6	0.481 ± 0.093 *
MIHA group	6	0.285 ± 0.068 *^,#^
*F*		29.41
*P*		0.00

* *p* < 0.05 *vs.* the sham-operated group, # *p* < 0.05 *vs.* the ICH group.

**Table 4:** The positive cell indexs (PCIs) of phospho-c-Jun NH_2_-terminal kinase (pJNK) in different groups at each time point ($\bar{x}$ ± s).

Groups	*n*	24 h	72 h	120 h
Sham-operated group	6	0.099 ± 0.030	0.110 ± 0.027	0.104 ± 0.026
ICH group	6	0.268 ± 0.024 *	0.320 ± 0.022 *	0.248 ± 0.031 *
MIHA group	6	0.198 ± 0.035 *^,#^	0.215 ± 0.028 *^,#^	0.182 ± 0.026 *^,#^
*F*		48.79	98.73	40.85
*P*		0.00	0.00	0.00

* *p* < 0.05 *vs.* the sham-operated group, # *p* < 0.05 *vs.* the ICH group.

**Table 5:** The relative value of the proteins (RVPs) of pJNK and c-Jun NH_2_-terminal kinase (JNK) at each time point determined by Western blotting ($\bar{x}$ ± s).

Groups	*n*	pJNK			JNK		
		24 h	72 h	120 h	24 h	72 h	120 h
Sham-operated group	6	0.390 ± 0.077	0.378 ± 0.083	0.383 ± 0.063	1.058 ± 0.082	1.125 ± 0.079	1.107 ± 0.088
ICH group	6	0.670 ± 0.084 *	0.779 ± 0.083 *	0.661 ± 0.070 *	1.062 ± 0.105	1.135 ± 0.090	1.099 ± 0.103
MIHA group	6	0.529 ± 0.079 *^,#^	0.606 ± 0.119 *^,#^	0.505 ± 0.076 *^,#^	1.078 ± 0.084	1.140 ± 0.088	1.106 ± 0.076
*F*		18.44	26.05	23.72	0.08	0.05	0.02
*P*		0.00	0.00	0.00	0.93	0.95	0.98

* *p* < 0.05 *vs.* the sham-operated group, # *p* < 0.05 *vs.* the ICH group.

## References

[1] Qureshi A.I., Mendelow A.D., Hanley D.F. (2009). Intracerebral haemorrhage. Lancet.

[2] Van Asch C.J., Luitse M.J., Rinkel G.J., van der Tweel I., Algra A., Klijn C.J. (2010). Incidence, case fatality, and functional outcome of intracerebral haemorrhage over time, according to age, sex, and ethnic origin: A systematic review and meta-analysis. Lancet Neurol..

[3] Hemphill J.C., Greenberg S.M., Anderson C.S., Becker K., Bendok B.R., Cushman M., Fung G.L., Goldstein J.N., Macdonald R.L., Mitchell P.H. (2015). Guidelines for the management of spontaneous intracerebral hemorrhage: A guideline for healthcare professionals from the American Heart Association/American Stroke Association. Stroke.

[4] Proust F., Leveque S., Derrey S., Tollard E., Vandhuick O., Clavier E., Langlois O., Fréger P. (2007). Spontaneous supratentorial cerebral hemorrhage: Role of surgical treatment. Neurochirurgie.

[5] Dey M., Stadnik A., Awad I.A. (2014). Spontaneous intracerebral and intraventricular hemorrhage: Advances in minimally invasive surgery and thrombolytic evacuation, and lessons learned in recent trials. Neurosurgery.

[6] Hibi M., Lin A., Smeal T., Minden A., Karin M. (1993). Identification of an oncoprotein and UV-responsive protein kinase that binds and potentiats the c-Jun activation domain. Genes Dev..

[7] Weston C.R., Davis R.J. (2007). The JNK signal transduction pathway. Curr. Opin. Cell Biol..

[8] Coffey E.T. (2014). Nuclear and cytosolic JNK signalling in neurons. Nat. Rev. Neurosci..

[9] Michel-Monigadon D., Bonny C., Hirt L. (2010). c-Jun N-terminal kinase pathway inhibition in intracerebral hemorrhage. Cerebrovasc. Dis..

[10] Sang Y.H., Liang Y.X., Liu L.G., Ellis-Behnke R.G., Wu W.T., So K.F., Cheung R.T. (2013). Rat model of intracerebral hemorrhage permitting hematoma aspiration plus intralesional injection. Exp. Anim..

[11] Zhang H., Ma X., Lu Y., Wang C. (2012). The intracereral hemorrhage model in rats. Guide China Med..

[12] Tournier C., Dong C., Turner T.K., Jones S.N., Flavell R.A., Davis R.J. (2001). MKK7 is an essential component of the JNK signal transduction pathway activated by proinflammatory cytokines. Genes Dev..

[13] Zhao Y., Spigolon G., Bonny C., Culman J., Vercelli A., Herdegen T. (2012). The JNK inhibitor D-JNKI-1 blocks apoptotic JNK signaling in brain mitochondria. Mol. Cell. Neurosci..

[14] Wang X., Zu J., Zan K., Shi H., Zhang Z., Kong L., Bao L., He Q., Zhou S., Cui G. (2014). The JNK inhibitor XG-102 protects against intracerebral hemorrhage. Acta Acad. Med. Xuzhou.

[15] Gong C., Hoff J.T., Keep R.F. (2000). Acute inflammatory reaction following experimental intracerebral hemorrhage in rat. Brain Res..

[16] Kaminska B., Gozdz A., Zawadzka M., Ellert-Miklaszewska A., Lipko M. (2009). MAPK signal transduction underlying brain inflammation and gliosis as therapeutic target. Anat. Rec..

[17] Benakis C., Bonny C., Hirt L. (2010). JNK inhibition and inflammation after cerebral ischemia. Brain Behav. Immun..

[18] Thornton P., McColl B.W., Cooper L., Rothwell N.J., Allan S.M. (2010). Interleukin-1 drives cerebrovascular inflammation via MAP kinase-independent pathways. Curr. Neurovasc. Res..

[19] Aronowski J., Zhao X. (2011). Molecular pathophysiology of cerebral hemorrhage: Secondary brain injury. Stroke.

[20] Bodmer D., Vaughan K.A., Zacharia B.E., Hickman Z.L., Connolly E.S. (2012). The Molecular Mechanisms that Promote Edema after Intracerebral Hemorrhage. Transl. Stroke Res..

[21] Katsuki H. (2010). Exploring neuroprotective drug therapies for intracerebral hemorrhage. J. Pharmacol. Sci..

[22] Hwang B.Y., Appelboom G., Ayer A., Kellner C.P., Kotchetkov I.S., Gigante P.R., Haque R., Kellner M., Connolly E.S. (2011). Advances in neuroprotective strategies: Potential therapies for intracerebral hemorrhage. Cerebrovasc. Dis..

[23] Keep R.F., Zhou N., Xiang J., Andjelkovic A.V., Hua Y., Xi G. (2014). Vascular disruption and blood-brain barrier dysfunction in intracerebral hemorrhage. Fluids Barriers CNS.

[24] Babu R., Bagley J.H., Di C., Friedman A.H., Adamson C. (2012). Thrombin and hemin as central factors in the mechanisms of intracerebral hemorrhage-induced secondary brain injury and as potential targets for intervention. Neurosurg. Focus.

[25] Wang X., Cui G., Meng W., Kong L., Bao L., He Q., Zhou S., Zu J., Liu Y. (2014). Changes of c-Jun N-terminal kinase in rat models of intracerebral hemorrhage and effect of argathoban on it. Chin. J. Neuromed..

[26] Huang F.P., Xi G., Keep R.F., Hua Y., Nemoianu A., Hoff J.T. (2002). Brain edema after experimental intracerebral hemorrhage: Role of hemoglobin degradation products. J. Neurosurg..

[27] Wan S., Zhan R., Zheng S., Hua Y., Xi G. (2009). Activation of c-Jun-N-terminal kinase in a rat model of intracerebral hemorrhage: The role of iron. Neurosci. Res..

[28] Sun W., Gould T.W., Newbern J., Milligan C., Choi S.Y., Kim H., Oppenheim R.W. (2005). Phosphorylation of c-Jun in avian and mammalian motoneurons *in vivo* during programmed cell death: An early reversible event in the apoptotic cascade. Neuroscience.

[29] Chen X.C., Fang F., Zhu Y.G., Chen L.M., Zhou Y.C., Chen Y. (2003). Protective effect of ginsenoside Rg1 on MPP^+^-induced apoptosis in SHSY5Y cells. J. Neural Transm..

[30] Lai B., Pu H., Cao Q., Jing H., Liu X. (2011). Activation of caspase-3 and c-Jun NH_2_-terminal kinase signaling pathways involving heroin-induced neuronal apoptosis. Neurosci. Lett..

[31] He Q., Bao L., Zimering J., Zan K., Zhang Z., Shi H., Zu J., Yang X., Hua F., Ye X. (2015). The protective role of (−)-epigallocatechin-3-gallate in thrombin-induced neuronal cell apoptosis and JNK-MAPK activation. Neuroreport.

[32] Zhang Q., Tang Q., Li X., Li J., Zhang L., Yan C., Cui Y. (2015). Effects of intracerebral hemorrhage and subsequent minimally invasive hematoma aspiration on expression of apoptosis related genes in rats. Int. J. Clin. Exp. Pathol..

[33] Yang G.Y., Betz A.L., Chenevert T.L., Brunberg J.A., Hoff J.T. (1994). Experimental intracerebral hemorrhage: Relationship between brain edema, blood flow, and blood-brain barrier permeability in rats. J. Neurosurg..

[34] Bederson J.B., Pitts L.H., Tsuji M., Nishimura M.C., Davis R.L., Bartkowski H. (1986). Rat middle cerebral artery occlusion: Evaluation of the model and development of a neurologic examination. Stroke.

